# Some flies do not play ping-pong

**DOI:** 10.1371/journal.pbio.3002152

**Published:** 2023-06-07

**Authors:** Arie Fridrich, Yehu Moran

**Affiliations:** 1 Gregor Mendel Institute of Molecular Plant Biology, Austrian Academy of Sciences, Vienna, Austria; 2 Department of Ecology Evolution and Behavior, Faculty of Science, Alexander Silberman Institute of Life Sciences, The Hebrew University of Jerusalem, Jerusalem, Israel

## Abstract

Genome integrity in animals depends on silencing of mobile elements by piRNAs. This Primer explores a new study in PLOS Biology that reveals recent evolutionary losses of key piRNA biogenesis factors in flies, highlighting adaptability by rapid shifts to alternative piRNA biogenesis strategies.

## Teaser

Genome integrity in animals depends on silencing of mobile elements by Piwi-interacting RNAs (piRNAs). A new study in this issue of *PLOS Biology* reveals recent evolutionary losses of key piRNA biogenesis factors in flies, highlighting adaptability by rapid shift to alternative piRNA biogenesis strategies.

**Fig 1:**
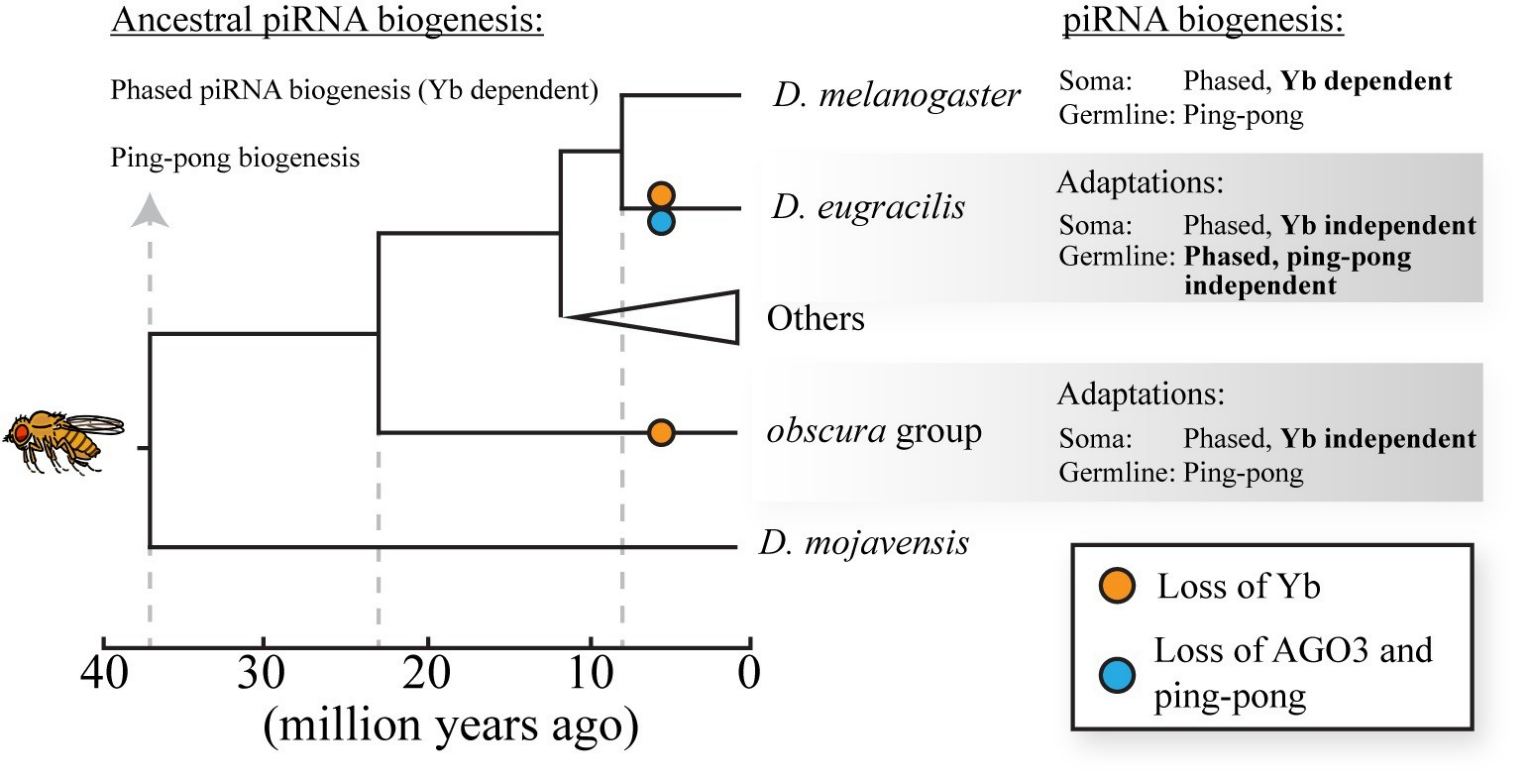
Conserved regulators of piRNA biogenesis are recurrently lost over short evolutionary timescales in close relatives of *D*. *melanogaster*. Germline ping-pong-mediated piRNA biogenesis and Yb-dependent somatic piRNA biogenesis are conserved among *Drosophila* species. Two independent losses of Yb occurred in *D*. *eugracilis* (which separated from *D*. *melanogaster* less than 10 million years ago) and in the obscura group (which separated from *D*. *melanogaster* approximately 20 million years ago). These losses were followed by adaptations to produce abundant somatic piRNAs in a Yb-independent manner. Additionally, in *D*. *eugracilis*, AGO3 was lost and the ping-pong piRNA biogenesis is absent from the germline. Germline piRNAs production in *D*. *eugracilis* shifted towards a ping-pong-independent mechanism.

The Piwi-interacting RNA (piRNA) pathway is crucial in animals for silencing transposable elements (TEs), prominent genomic parasites, by directing their transcriptional and posttranscriptional suppression [1]. Despite being highly conserved in almost all lineages stemming from the last common ancestor of animals [2], alterations in the biogenesis of piRNA components have been observed in distantly related species. However, the immediate adaptive responses of species to these alterations remain largely unknown, and studying them is challenging as deleting these components in model organisms often leads to sterility. In this issue of *PLOS Biology* [3], Chary and Hayashi have identified 2 species of *Drosophila* flies that have undergone radical and recent alterations in their piRNA biogenesis (**Fig 1**), providing a unique opportunity to investigate adaptation on a much shorter evolutionary timescale.

To generate piRNAs in the range of 24–30 nucleotides that target TEs, most animals process transcripts from single-stranded piRNA precursors that exhibit sequence homology to the TEs that should be targeted. Since the discovery of precursor piRNA genomic clusters [4], 2 major modes of biogenesis have been revealed. The phased mode enables the cleavage of genomic piRNA precursor transcripts using a conserved metazoan endonuclease, which enables sequential cleavage of the long piRNA precursor into multiple piRNAs [5,6]. Following this step, primary TE antisense piRNAs are carried by Piwi into the nucleus to silence the chromatin of transcribed TEs. In the second mode of piRNA biogenesis that is known as “ping-pong,” piRNA precursors are cleaved by the Piwi paralogs themselves. These cleavages generate the 5′ ends of new piRNAs that are then loaded into a new, empty Piwi paralog. In this process, Aubergine (Aub) triggers the loading of AGO3 and vice versa, hence the name “ping-pong.” Thus, an efficient silencing cycle is established [7] where sense piRNA precursors as well as TEs themselves enable biogenesis of TE antisense piRNAs. Additionally, AGO3-mediated cleavage of TE antisense precursors can also trigger biogenesis of phased antisense piRNAs.

It was long believed that the ping-pong cycle was essential for piRNA biogenesis in the germline of flies ovaries, as mutations in the pathway resulted in sterility in *Drosophila melanogaster*, the major lab model species where the piRNA pathway was originally characterized. However, the current study by Chary and Hayashi found that a closely related species, *Drosophila eugracilis*, which diverged from *D*. *melanogaster* less than 10 million years ago, had lost the ping-pong cycle [3] (**Fig 1**). This raises important questions about how *D*. *eugracilis* has adapted to cope with the loss of this vital biogenesis pathway. Interestingly, the authors found that despite the loss of the ping-pong cycle, piRNA abundance remained high in *D*. *eugracilis*, suggesting that loss of a need to silence transposons did not drive the loss of this pathway. The authors show that instead *D*. *eugracilis* shifted the balance in favor of phased piRNA biogenesis [3]. A remaining intriguing question is what was the molecular platform that enabled the system to shift to fully ping-pong independent piRNA biogenesis.

It is tempting to hypothesize that *D*. *eugracilies* may have exploited an existing ping-pong-independent piRNA biogenesis pathway that operates in the somatic ovarian tissues of other *Drosophila* species, including *D*. *melanogaster*. In these tissues, a unique type of Gypsy retrotransposons that encode an envelope component (env) must be silenced to prevent the production of infectious particles that can invade the neighboring germline. The piRNA biogenesis factor Yb plays a crucial role in repressing these TEs in the somatic tissue and is vital for the establishment of nuage-like cytoplasmic perinuclear bodies, where piRNA biogenesis factors are recruited [8,9]. In Yb mutants of *D*. *melanogaster*, the piRNA processing bodies are lost, TEs are reactivated, and the flies become sterile. Despite its conservation across flies, the authors identified 2 independent losses of Yb (**Fig 1**), one in the more distantly related lineage of *Drosophila* species, called the “*obscura* group” (represented in the current study mainly by *D*. *pseudoobscura*) and the other in *D*. *eugracilis*. However, both lineages maintain intact nuage-like perinuclear bodies for piRNA processing and abundantly produce piRNAs [3]. It is extraordinary that an essential lineage-specific piRNA biogenesis factor was lost twice independently in closely related species. Furthermore, the finding that *D*. *eugracilis*, which is closely related to *D*. *melanogaster*, lost both Yb and the ping-pong biogenesis components highlights the remarkable adaptive capacity of this species to cope with radical alterations in the piRNA biogenesis pathway over short evolutionary timescales. Therefore, the piRNA system in these species provides an excellent molecular model to investigate the evolution of rapid adaptive responses to drastic changes in a host’s genome defense systems over short evolutionary timescales.

The majority of animals, vertebrates and invertebrates, including *D*. *melanogaster* depend on ping-pong piRNA biogenesis in their germline, which probably represent an ancestral state. Pan-arthropod analysis revealed that multiple arthropod lineages with somatic piRNAs have operating ping-pong mechanism in the soma [10]. Why the *Drosophila* genus evolved ping-pong-independent somatic piRNA biogenesis is a mystery and demonstrates the evolutionary dynamic nature of the piRNA pathway. Their fertility is clearly dependent on somatic phased piRNA biogenesis facilitated by Yb. The findings in *D*. *eugracilis* and the *obscura* group of the current study demonstrate that adaptations of the piRNA system can also occur over much shorter evolutionary times than previously observed. What drove those rapid changes remains to be described; however, it is tempting to speculate that changes in the TE contents and their expression domain might have contributed as part of an “arms-race” between the host and its genomic parasites. Further studies are needed to fully understand the evolution of the piRNA system and its adaptive responses to changes over short evolutionary timescales and the current study takes an important step forward in this direction.
